# Validation of a mobility item bank for older patients in primary care

**DOI:** 10.1186/1477-7525-10-147

**Published:** 2012-12-05

**Authors:** Julio Cabrero-García, Juan Diego Ramos-Pichardo, Carmen Luz Muñoz-Mendoza, María José Cabañero-Martínez, Lorena González-Llopis, Abilio Reig-Ferrer

**Affiliations:** 1 Department of Nursing, University of Alicante, Ctra. San Vicente s/n, San Vicente del Raspeig, Alicante, 03015, Spain; 2 Department of Health, Alcoy, Alicante, Spain; 3 Department of Health Psychology, University of Alicante, Alicante, Spain

**Keywords:** Mobility, Differential item functioning, Rasch analysis, Gender differences, Older people, Primary care, Item bank

## Abstract

**Background:**

To develop and validate an item bank to measure mobility in older people in primary care and to analyse differential item functioning (DIF) and differential bundle functioning (DBF) by sex.

**Methods:**

A pool of 48 mobility items was administered by interview to 593 older people attending primary health care practices. The pool contained four domains based on the International Classification of Functioning: changing and maintaining body position, carrying, lifting and pushing, walking and going up and down stairs.

**Results:**

The Late Life Mobility item bank consisted of 35 items, and measured with a reliability of 0.90 or more across the full spectrum of mobility, except at the higher end of better functioning. No evidence was found of non-uniform DIF but uniform DIF was observed, mainly for items in the changing and maintaining body position and carrying, lifting and pushing domains. The walking domain did not display DBF, but the other three domains did, principally the carrying, lifting and pushing items.

**Conclusions:**

During the design and validation of an item bank to measure mobility in older people, we found that strength (carrying, lifting and pushing) items formed a secondary dimension that produced DBF. More research is needed to determine how best to include strength items in a mobility measure, or whether it would be more appropriate to design separate measures for each construct.

## Background

Physical function is a central component of health status and quality of life [1]. In addition to measuring physical function with fixed length scales such as the Health Assessment Questionnaire [2] or the subscale of physical functioning of the Medical Outcomes Study Short Form-36 (PF-10) [3], it can also be measured using item banks based on item response theory (IRT) models [4,5]. In some of these item banks, physical function is measured as a two-dimensional construct consisting of mobility and upper extremity function [6,7], although in others a unidimensional solution has been considered more appropriate [8,9]. Nevertheless, it is true that the latter is not sufficiently robust for certain health conditions [9]. The majority of these physical function measures are aimed at assessing health outcomes in patients with chronic diseases or in rehabilitation contexts [6-10]. However, there are no specific measures to assess physical function in community dwelling older people, with the exception of the Late-life Function and Disability Instrument [11,12].

Measuring physical function - mainly mobility rather than upper extremity function - in older people is doubly useful as physical function is a strong predictor of disability, institutionalisation and death and is also a primary outcome, more proximal than disability, in longitudinal and clinical trials aimed at explaining or preventing disability [13,14]. Due to the scarcity and importance of late life mobility measures, the first of the two objectives of this paper is to present the development and validation of an item bank to measure mobility in community dwelling older people, using IRT methods. Items in the item bank were based on International Classification of Functioning, Disability and Health (ICF) mobility indicators [15]. Consequently, neither upper extremity function items nor disability (in activities of daily living) items were included.

In addition, significant gender differences in mobility have been observed, in the sense that women present a poorer function [16,17]. These differences are not uniform across the mobility domains, but are greater in the carrying, lifting and pushing domains than in the walking and moving domains [17-22]. However, psychometric studies analysing gender differential item functioning or DIF—namely, depending on construct level, whether the probability of responding to an item differs for the compared groups—have not yielded any relevant or systematic findings, except that most DIF effects are cancelled at the level of aggregate score [8,9,12,23,24]. For example, nine items in the physical function computerised adaptive testing version of the European Organisation for Research and Treatment of Cancer Quality of Life Questionnaire-Core 30 showed gender DIF, but DIF cancellation occurred because the DIF observed was in opposite directions: walking and moving items were more demanding for men whereas carrying, lifting and pushing items were more demanding for women [24].

However, although DIF cancellation can be secured in a fixed measure or even in an entire item bank, this is not the case in adaptive measures created from this latter [23,25]. In a standard DIF analysis, an internal criterion—total score or an estimate based on total score—is used as a conditioning variable and then each item is individually studied for DIF [23]. However, it is also possible to study a bundle of items simultaneously rather than separately, and by analysing item bundles it becomes possible to test the DIF amplification hypotheses, i.e., whether items depending on a common secondary dimension have DIF effects, significant or nonsignificant, which accumulate at the level of item domain or bundle (differential bundle functioning or DBF) [26,27]. Accordingly, the second objective of this study was to examine whether mobility domains form secondary dimensions containing items that present DBF.

Therefore, the two objectives of this paper are to present the development and validation of an item pool to measure mobility in older people and to analyse differential item and bundle functioning across gender.

## Methods

### Study population

The data presented in this article have been taken from the baseline of a longitudinal study on mobility measures as predictors of adverse health outcomes. People considered eligible for participation in the study comprised those over 69 years old attending five primary health care centres in the Autonomous Region of Valencia (Spain). Those patients who produced more than three errors (four if they were illiterate) in the Short Portable Mental Status Questionnaire [28], had serious communication problems or were considered too weak to participate in physical performance tests, were excluded. Sampling was consecutive: all eligible patients from one day of each week during the period November 2006 to October 2007 were selected. Of the 700 eligible patients, 593 gave informed consent and comprised the study sample. No statistically significant differences between participants and non-participants were observed for age or sex. The participants gave their informed consent and the study was approved by the corresponding authorities of the health centres involved.

### Measures

#### Late life mobility item bank (LLM-IB)

A pool of 104 mobility items was selected from the literature and a panel of experts (two physicians, four nurses and three psychologists) assessed their relevance and suitability for older people, and also classified them into four domains based on three ICF categories of mobility: changing and maintaining body position (BP), carrying, lifting and pushing (CLP), walking (Walking) and going up and down stairs (UDS). Walking and UDS were considered separately and items relating to moving around using transportation were not included. The relevance of the activities included was also evaluated by three focus groups of older people. As a result of the above, 48 items were selected and their ease of understanding was assessed in 17 cognitive interviews. No items were eliminated, but modifications were made to various item statements. The item stem posed the question in terms of ability, in the present tense and made no reference to health, with a rating scale of four response categories: no difficulty, some difficulty, much difficulty and unable to do. Scores were scaled measuring mobility limitation: the higher the score, the worse the function.

#### Other mobility measures

PF-10 and the Short Physical Performance Battery (SPPB) were used as external criteria for the mobility item bank. PF-10 is a 10-item self-report measure based mainly on lower extremity mobility [3,29]. The SPPB battery objectively assesses physical function of the lower extremities. It consists of three tests: balance, gait speed and chair stand. It has demonstrated excellent reliability, predictive validity and sensitivity to clinically important change and has been recommended for objectively measuring mobility limitations [14,30].

#### Biodemographic, clinical and disability measures

Biodemographic variables included body mass index (kg/ m^2^), age, sex, education and living arrangements. Cognitive function was evaluated using the Short Portable Mental Status Questionnaire [28]. Symptoms of depression were evaluated with the Geriatric Depression Scale [31]. Morbidity was measured by the presence or absence of the following medical diagnoses: hypertension, rheumatoid arthritis, osteoarthritis, myocardial infarction, angina pectoris, congestive heart failure, diabetes, cancer, chronic pulmonary disease, stroke, hip fracture, Parkinson’s disease, and claudication [32,33]. Finally, subjects were asked whether they needed the help of another person to complete any of the following activities: eating, toileting, bathing, dressing and transferring (ADL dependence).

### Procedure

Measurements were collected at the primary health care centres, but not during the subject’s medical appointment. The SPPB was administered by trained observers, who also recorded height and weight, morbidity was reported by the doctors caring for the patients who participated in the study and the other measures were completed in an interview situation, conducted by the same observers. Reliability of the mobility item pool and the SPPB was assessed in a pilot study. Using an interval of 15 days and a sample size of n = 62, the intra-class correlation coefficient for intra-rater reliability was 0.90 for the entire item pool, with a range of 0.60 - 0.90 for each of the items. Intra-class correlation coefficient for SPPB intra-rater reliability was 0.80 (n = 62) and for inter-rater reliability, 0.88 (n = 30).

### Data analysis

The main analyses consisted of examining DIF and DBF and calibrating the item pool using the Rasch rating scale model (RSM) [34]. Prior to this however, we performed a descriptive analysis of the items and examined the three assumptions common to IRT models: monotonicity, unidimensionality and local independency. Unidimensionality is also an assumption for standard DIF analysis. Since the unidimensionality of a measure in a population does not ensure its unidimensionality in subpopulations [35], this aspect was also analysed separately in the subsamples of women and men. DIF/DBF analysis was performed before calibrating the item pool to avoid confusing item DIF with item misfit

#### IRT assumptions

TestGraf [36] was used to analyse whether the items had a monotonic relation with the construct and if each response category had a maximum probability of being selected over a unique interval of the scale. TestGraf estimates and displays the characteristic response curves by means of the nonparametric regression method known as kernel smoothing. To examine the unidimensionality of the item pool, we tested confirmatory, single and bifactor models with factor analysis methods suitable for ordinal data, namely analysis of polychoric correlation matrices using a diagonally weighted least squares estimator [4,37,38]. We specified four group factors in the bifactor model, one for each mobility item pool domain. These analyses were performed for the entire sample and also for the male and female sub-samples. To measure goodness-of-fit of the models, we selected the Comparative Fit index (CFI), the Tucker Lewis Index (TLI), the root-mean-square error of approximation (RMSEA) and the standardised root mean square residual (SRMR) indices [4]. The cut-off values were as follows: 0.95 for TFI and CFI, 0.08 for RMSEA and 0.06 for SRMR [4,39]. For the bifactor models, we also estimated the proportion of variance explained by group and general factors, together with differences between common factor loadings for the single and bifactor models [38]. Moreover, residual correlations were calculated for the single factor models and r > 0.2 was selected as the cut-off for determining the presence of local dependency [4]. LISREL was used for these analyses [37].

#### Differential item and differential bundle functioning analysis by sex

The simultaneous item bias test (SIBTEST) framework was used to assess DIF. SIBTEST is a nonparametric method which enables DIF to be tested both at item and item bundle levels [40]. An item bundle is a subset of substantively homogeneous or statistically dimensionally homogeneous items which measure a dimension secondary to the dominant dimension measured for the entire pool [40]. In this study, the bundles consisted of the four mobility item pool domains. SIBTEST permits formal statistical testing of item DIF and DBF, and a magnitude measure, β. The β scale is the probability scale for single item analysis and the expected score scale for bundle analysis. Bundle β is simply the sum of item β for each of the bundle items [41].

Standard item DIF analysis uses an internal criterion, total score or a latent ability estimate, as a conditioning variable [35]. Since the conditioning variable should not have any items with significant DIF, a prior purification stage was implemented before the definitive item DIF analysis. The two types of DIF, uniform and non-uniform, were analysed: the Poly-SIBTEST (SIBTEST for ordinal data) was used to assess uniform DIF and the Crossing-SIBTEST for non-uniform DIF [42,43]. As only binary data can be analysed with the Crossing-SIBTEST, categories on the rating scale were combined as follows: no difficulty vs. the rest. Items were flagged for DIF if P < 0.05, using Bonferroni correction for multiple testing. We also conducted a sensitivity analysis of DIF: for uniform DIF we assessed differences between item locations produced in a Rasch RSM analysis, for each group, using t-tests; for non-uniform DIF, we used TestGraf to graphically examine the differences between the item response curves for each group.

To examine DBF, which is the cumulative effect of significant and nonsignificant item DIF across the item domain, we used two external criteria as conditioning variables, PF-10 and SPPB. Since PF-10 is a self-report measure, this criterion is the closest to the mobility item pool. However, SPPB, which is a mobility standard based on objective performance, can be useful for detecting pervasive DIF produced by self-report measures. Analysing DBF entails analysing item DIF, and therefore the results of the latter are also given.

#### IRT analysis: Rasch RSM

The item pool was calibrated using the Rasch RSM, the simplest Rasch model for polytomous items [44]. RSM allows items to vary in their level of difficulty but assumes that all items are equally discriminant and share the same rating scale structure [44]. Due to its more restrictive nature, it is robust for small or medium sized samples and is likely to provide more generalisable results [45]. In the RSM, response categories (K) are assigned intersection parameters (K – 1 intersection parameters or thresholds) which are considered equal across items, and an item location is described by a single parameter that indicates the difficulty or ease of the item relative to category thresholds [34]. The RSM enables estimates of item location, category thresholds and subject score to be placed on the same metric. The fit of data to the RSM was assessed with infit and outfit mean square error statistics, using a cut-off of <0.6 or >1.4 for possible item deletion [9,44]. Item deletion was implemented sequentially and concluded once none of the remaining items showed misfit. To assess the accuracy of the final item bank, the test information function and its reciprocal [46] the standard error function, were calculated. The person reliability index (analogous to Cronbach’s alpha, but excluding extreme scores [47]) was also calculated. To examine item bank coverage and suitability for the sample, item difficulties and person scores were plotted together, centering the scale on zero logits—the average difficulty of items. Finally, the mobility item bank and the PF-10 items were grouped according to their response options and then co-calibrated onto one common construct (mobility). We used the same pivot anchor for both rating scales: the step from “no difficulty” (or “no limitation”) to the next [48]. WINSTEPS was used for these analyses [49].

#### Missing data

All of the analyses except the RSM analysis were performed using imputed data obtained through matching, employing the PRELIS (LISREL) Impute Missing Value dialog box. For the RSM analysis, Joint Maximum Likelihood was implemented as the estimation method. This method does not require missing data to be imputed but considers such data ignorable.

## Results

Table 1 presents the demographic and clinical characteristics of the subjects. 

**Table 1:** Sample characteristics

	**Sample (n = 593)**	**Male (n = 252)**	**Female (n = 341)**
**Age**			
Mean (SD)	76.53 (4.81)	76.73 (4.78)	76.39 (4.84)
Median	76	76	76
Range	70 – 98	70 - 96	70 – 98
**Gender** (%)			
Female	57.50		
**Education** (%)			
Unable to read/write	16.69	9.13	22.29
Able to read/write	58.52	59.92	57.48
Primary	16.86	19.44	14.97
Secondary or higher	7.93	11.51	5.28
**Living** (%)			
Couple	60.03	79.37	45.75
Family	14.84	8.73	19.35
Alone	25.13	11.90	34.90
**Morbidity Index** (%)			
0-1	21.92	29.37	16.42
2	23.44	22.62	24.05
+2	54.64	48.01	59.53
Mean (SD)	2.90 (1.78)	2.65 (1.78)	3.08 (1.76)
**Body Mass Index**			
Mean (SD)	29.12 (4.28)	28.14 (3.81)	29.85 (4.46)
**Cognitive Status**			
SPMSQ: mean (SD)	1.58 (1.12)	1.35 (1.01)	1.75 (1.11)
**Functional Status**			
ADL dependence:%	6.41	3.57	8.50
SPPB: mean (SD)	8.35 (2.73)	9.17 (2.41)	7.74 (2.80)
PF-10: mean (SD)	67.90 (26.35)	76.38 (22.45)	61.59 (27.29)
	65.70 (29.80)^*^	73.30 (27.00) ^*^	63.10 (25.10) ^*^

* Reference population values of Spanish people aged 70 and over [50].

*SPMSQ* Short Portable Mental Status Questionnaire.

*ADL* Activities of Daily Living.

*SPPB* Short Physical Performance Battery.

*PF-10* Subscale of physical functioning of the Medical Outcomes Study Short Form-36.

### Descriptive analysis of the item pool

Three items returned percentages for the first response option (“no difficulty”) of 90% or more, the item-test correlations ranged between 0.53 and 0.83 and percentages of missing responses per item were less than 5% in all cases with the exception of two which were slightly higher.

#### IRT asumptions

**Figure 1:**
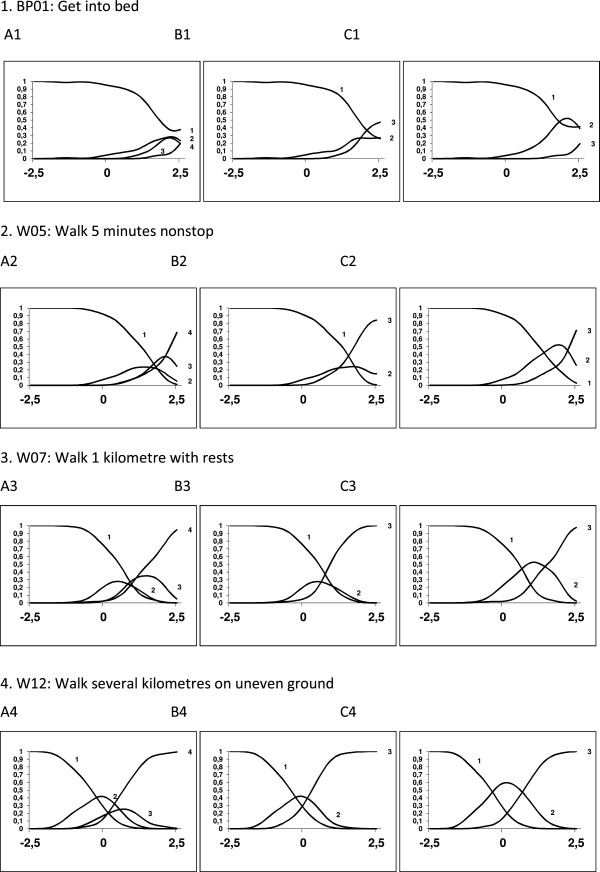
**Four examples of different characteristic option curves.** Characteristic option curves sorted by degree of difficulty (top-down: lowest to highest difficulty) and by type of rating scale (from left to right: A = No difficulty; Some difficulty; Much difficulty; Unable to do. B = No difficulty; Some difficulty; Much difficulty + Unable to do. C = No difficulty; Some difficulty + Much difficulty; Unable to do). The first item was very easy and was unsuitable for the sample, although with rating scale type C it showed good behaviour. The second and third items only presented the desired features with rating scale type C, while the fourth one showed the desired features with rating scale types B and C.

The item response curves had a monotonic relation with the construct for all the items; however, the slopes of three items were not steep enough (items previously identified with percentages > 90% in the first response option). As regards the characteristic response curves, for the majority of the items the intermediate option curves (“some difficulty”, “much difficulty”) lacked a maximum over a unique interval of the scale. Therefore, we examined two possibilities: combining both intermediate options or combining the last two options, i.e., no difficulty, some + much difficulty and unable to do, vs. no difficulty, some difficulty, much difficulty + unable to do. The first solution was clearly better since the curves for all the items would then have a maximum over a unique interval of the scale, whilst in the second solution, the curves for the intermediate option would lack a maximum for the majority of the items. Figure 1 shows examples of these curves for four items with each of the three rating scales. Consequently, we eliminated the three items which were flagged and recoded the rating scale for the successive analyses into three categories: no difficulty, some/much difficulty and unable to do.

Table 2 gives the confirmatory factor analysis results both for the entire sample and separately for men and women. Item loadings and fit indices of the single factor model supported a unidimensional interpretation of the item pool. Furthermore, the results for the bifactor model indicated that the influence of the domains (group factors) did not distort this interpretation: the differences between common factor loadings in the bifactor model and the single-factor model did not exceed 0.10, with a median of 0.01; the group factors explained only 9.29% of variance vs. 66.43% for the common factor, and no item had a higher loading for the group factor than for the common factor. This pattern of results was repeated in analyses by sex, although the influence of the CLP group factor was higher in men. 

**Table 2:** Model fit statistics for confirmatory factor analyses

**Models**	**CFI**	**NNFI (TLI)**	**RMSEA**	**SRMR**	**Satorra**-**Bentler χ**^**2**^	**df**
Single	.99	.99	.072	.056	3816.72^*^	945
Bifactor	1.00	.99	.048	.040	2126.52^*^	900
Single (female)	.99	.99	.072	.061	2475.04^*^	902
Bifactor (female)	.99	.99	.051	.047	1599.65^*^	858
Single (male)	.99	.99	.072	.083	2079.19^*^	902
Bifactor (male)	.99	.99	.055	.067	1506.36^*^	858
Standard cutoff val.	> .95	> .95	< .06	< .08		

*CFI* Comparative Fit Index.

*NNFI (TLI)* Non Normed Fit Index (Tucker Lewis Index).

*RMSEA* Root Mean Square Error of Approximation.

*SRMR* Standardized Root Mean Square Residual.

*df* degree of freedom.

* p < 0.01.

All the residual correlations for the single-factor model were lower than 0.2, except one which was 0.21 (the items “Sitting, bend over to pick something up” and “Standing, bend down to pick something up”); consequently, we considered that there were no local dependencies in the item pool.

### DBF and DIF analysis

Standard DIF analysis with the purified conditioning variable flagged the same items with significant DIF as the DIF analysis with no purified conditioning variable. Table 3 gives a summary of DIF results. No item was flagged for non-uniform DIF, but there was evidence of uniform DIF: one item from the Walking domain (W11), three from the BP domain (BP10, BP14, BP15) and two from the CLP domain (CLP03, CLP05) were flagged for significant DIF. No item from the UDS domain was flagged for significant DIF. Furthermore, most of the Walking domain items presented negative (nonsignificant) DIF and all the CLP domain items showed positive (significant or nonsignificant) DIF.

**Table 3:** **Differential item functioning** (**DIF**) **and differential bundle functioning** (**DBF**) **results**

	**Item Wording**			**External criteria**			
		**Internal criterion**		**PF**-**10**		**SPPB**	
		**Beta**	**p**	**Beta**	**p**	**Beta**	**p**
**Walking** (**W**)							
W01	Walk around the house	-.01	.75	-.02	.41	-.03	.32
W02	Walk without losing balance	.02	.56	.03	.38	.01	.71
W03	Walk without tripping	.05	.13	.07	.03	.07	.07
W04	Walk outdoors	-.06	.06	-.05	.25	-.08	.07
W05	Walk 5 minutes nonstop	-.04	.23	-.03	.40	-.05	.19
W06	Cross the road	.04	.06	.05	.11	.01	.82
W07	Walk 1 kilometre with rests	-.08	.07	-.04	.43	-.11	.03
W08	Walk on a slippery surface	.01	.80	.06	.19	.01	.87
W09	Walk 15 minutes nonstop	-.07	.08	-.09	.04	-.12	.02
W10	Walk 5 minutes at a fast pace	.14	.01	-.11	.05	-.14	.01
W11	Walk 2 kilometres nonstop	-.**21**	.**00**	-.14	.01	-.17	.00
W12	Walk several kilometres on uneven ground	-.03	.58	.04	.40	-.01	.93
W13	Walk half an hour at a fast pace nonstop	-.14	.01	-.04	.50	-.07	.30
W14	Climb a steep hill	-.02	.59	.07	.07	.09	.06
W15	Run a short distance	-.05	.05	.02	.68	-.01	.91
W16	Run one and a half kilometres	-.08	.06	.04	.43	.02	.71
	DBF			-.14		-.58	
	Average DBF (DBF/n items)			-.01		-.04	
**Up and down stairs** (**UDS**)							
UDS01	Climb 4 or 5 steps, using handrails	.05	.09	.03	.28	.05	.19
UDS02	Step up and down from a curb	.01	.76	.06	.09	.02	.58
UDS03	Climb 4 or 5 steps, without handrails	.06	.18	.10	.05	.06	.30
UDS04	Go down 4 or 5 steps, using handrails	.05	.11	.04	.17	.03	.34
UDS05	Get on and off a bus	.05	.17	.11	.01	.06	.14
UDS06	Go down 4 or 5 steps, without handrails	.06	.15	.13	.01	.10	.07
UDS07	Go up 1 flight of stairs, without handrails	.02	.74	.10	.06	.08	.19
UDS08	Go down 1 flight of stairs, using handrails	.05	.11	.06	.07	.06	.10
UDS09	Climb stairs carrying little weight	-.05	.17	-.04	.29	-.02	.61
UDS10	Go up 3 flights of stairs, using handrails	.02	.60	.06	.13	.07	.14
UDS11	Go down 3 flights of stairs, using handrails	.01	.70	.03	.42	.05	.32
	DBF			.**68**		.56	
	Average DBF (DBF/n items)			.06		.05	
**Body Position** (**BP**)							
BP01	Get into bed†	-	-	-	-	-	-
BP02	Turn over in bed	.01	.91	.05	.29	.06	.15
BP03	Sit down on a couch	.02	.50	.05	.16	.02	.54
BP04	Sitting, bend over to pick something up	-.04	.32	-.02	.66	.01	.81
BP05	Remain seated for 10 minutes without back rest	.07	.05	.**13**	.**00**	.**16**	.**00**
BP06	Sit up in bed, being lied down	-.03	.47	-.01	.74	-.00	.97
BP07	Sit down and stand up from a chair†	-	-	-	-	-	-
BP08	Stand up from a low, soft couch	.10	.03	.09	.04	.08	.11
BP09	Pick up a chair†	-	-	-	-	-	-
BP10	Reach overhead while standing	.**19**	.**00**	.**22**	.**00**	.**26**	.**00**
BP11	Turn around while standing	.07	.09	.08	.04	.09	.04
BP12	Remain standing for 10 minutes	-.03	.50	-.01	.83	.00	.98
BP13	Standing, bend down to pick something up	-.04	.37	.01	.80	.01	.80
BP14	Get up from the floor from lying on your back	.**20**	.**00**	.**24**	.**00**	.**23**	.**00**
BP15	Kneel down	.**29**	.**00**	.**32**	.**00**	.**32**	.**00**
BP16	Get into and out of a car	.03	.53	.08	.06	.08	.06
	DBF			**1**.**23**		**1**.**32**	
	Average DBF (DBF/n items)			.09		.10	
**Carrying**, **Lifting and Pushing** (**CLP**)							
CLP01	Push or pull a large object	.07	.09	.12	.01	.13	.01
CLP02	Move or drag a bed	.11	.02	.13	.00	.**16**	.**00**
CLP03	Lift 4 or 5 kg from the floor	.**17**	.**00**	.**22**	.**00**	.**25**	.**00**
CLP04	Turn over a mattress	.10	.04	.**14**	.**00**	.**18**	.**00**
CLP05	Change gas bottle	.**23**	.**00**	.**22**	.**00**	.**23**	.**00**
	DBF			.**83**		.**95**	
	Average DBF (DBF/n items)			.17		.19	

DIF: in bold, significant values p < 0.002; DBF: in bold, significant values p < 0.01.

† Items excluded after Testgraf analysis.

DIF analysis with the two external criteria as conditioning variables produced very similar results: most of the CLP domain items showed significant item DIF, and the BP domain items which were flagged for significant item DIF were the same as those which had been flagged by the standard item DIF analysis. The results of DBF analysis also coincided with the two external criteria: three domains presented DBF (the Walking domain was the exception), but the magnitude was only substantial and consistent across the items in the CLP domain (Table 3).

We have decided to delete items that were consistently (by the three criteria) flagged for significant DIF, but we kept one of them (BP14) because it measured in the highest level of the construct.

### Rasch RSM analysis

**Figure 2:**
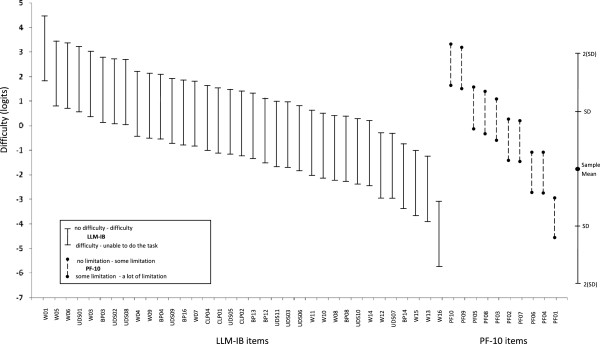
**Coverage and suitability of the item bank for the sample.** Difficulties of the items and scores of subjects are plotted together. On the left: items from the Late Life Mobility item bank (LLM-IB), on the right: items from the PF-10 subscale. W: items from the walking domain, UDS: items from the going up and down stairs domain, BP: items from the changing and maintaining body position domain, CLP: items from the carrying, lifting and pushing domain and PF: items from the PF-10 subscale.

**Figure 3:**
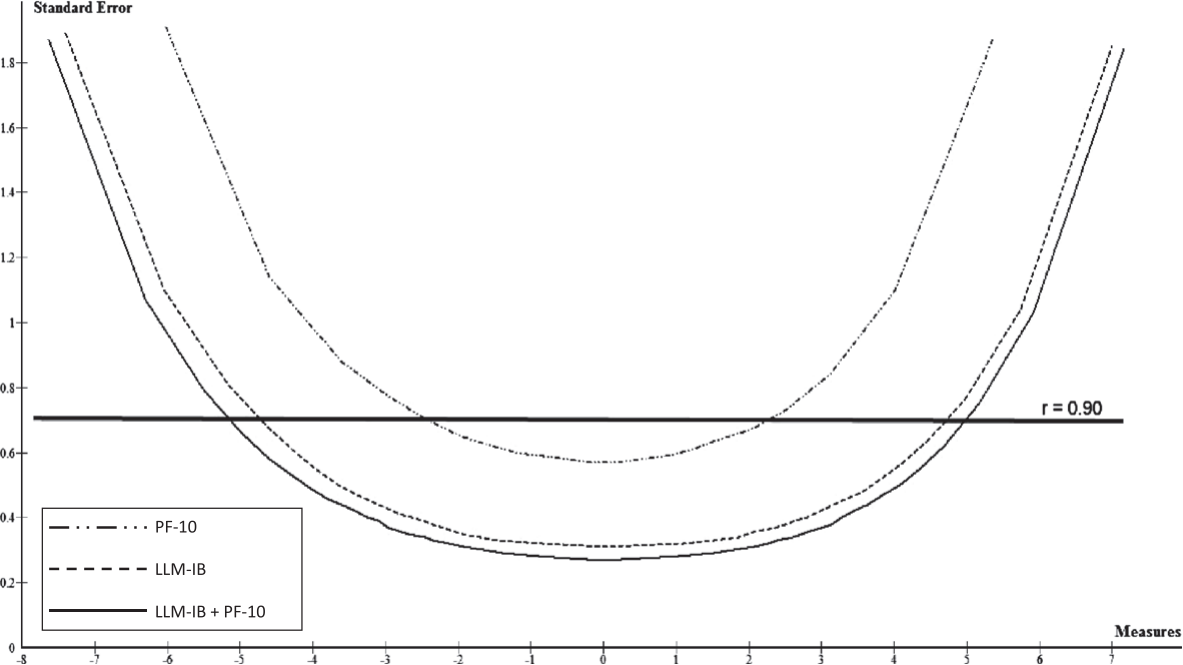
Standard error functions of the Late Life Mobility item bank (LLM-IB), PF-10 subscale and Late Life Mobility item bank + PF-10 subscale.

Six items, one from the Walking, one from the UDS and four from the BP domains were iteratively eliminated because of misfit. Table 4 shows the category thresholds, item locations and mean square error statistics for the remaining 35 items (15 Walking items, 10 UDS items, 7 BP items and 3 CLP items). Item pool coverage and accuracy was satisfactory throughout the entire continuum of mobility, with the exception of the upper level of capacity, which corresponds to more demanding activities than running 500 m without difficulty or performing vigorous activities (Figures 2 and 3). 6.7% of people obtained the lowest score (greatest capacity or least mobility limitation) and no person received the maximum score. The person reliability index was 0.95. Figures 2 and 3 also show the results for co-calibration of LLM-IB and PF-10.

**Table 4:** **Summary of Rasch rating scale analysis and confirmatory factor analysis** (**loadings**)

		**Confirmatory factor analysis**						**Rasch rating scale analysis**		
		**Single Factor**	**Bi**-**factor model**					**Location (Error)**	**Infit MNSQ**	**Outfit MNSQ**
			**G**	**g1**	**g2**	**g3**	**g4**			
**Walking** (**W**)										
W01	Walk around the house	.88	.86	.27				3.15 (.15)	.88	.54
W02	Walk without losing balance**	.81	.80	.16				-	-	-
W03	Walk without tripping	.78	.78	.06				1.71 (.11)	1.02	1.47
W04	Walk outdoors	.89	.86	.29				.91 (.10)	.78	.72
W05	Walk 5 minutes nonstop	.83	.79	.33				2.12 (.12)	1.13	.73
W06	Cross the road	.87	.87	.17				2.02 (.12)	.91	.77
W07	Walk 1 kilometre with rests	.89	.83	.40				.50 (.09)	.98	.81
W08	Walk on a slippery surface	.82	.82	.14				- .84 (.09)	.95	1.29
W09	Walk 15 minutes nonstop	.88	.81	.46				.84 (.10)	1.03	.95
W10	Walk 5 minutes at a fast pace	.87	.80	.44				- .74 (.09)	1.22	1.00
W11	Walk 2 kilometres nonstop	.90	.84	.43				- .66 (.09)	1.03	.83
W12	Walk several kilometres on uneven ground	.88	.85	.27				- 1.53 (.08)	.90	.84
W13	Walk half an hour at a fast pace nonstop	.88	.81	.46				- 2.47 (.09)	1.04	.90
W14	Climb a steep hill	.81	.80	.20				- 1.06 (.09)	.89	1.29
W15	Run a short distance	.88	.86	.26				- 2.31 (.09)	1.08	.89
W16	Run one and a half kilometres	.83	.80	.30				- 4.30 (.10)	1.29	.97
**Up and down stairs** (**UDS**)										
UDS01	Climb 4 or 5 steps, using handrails	.86	.83		.40			1.89 (.11)	.87	.72
UDS02	Step up and down from a curb	.86	.87		.11			1.42 (.11)	.81	.80
UDS03	Climb 4 or 5 steps, without handrails	.93	.93		.16			- .31 (.09)	.97	.77
UDS04	Go down 4 or 5 steps, using handrails**	.80	.75		.54			-	-	-
UDS05	Get on and off a bus	.88	.89		.03			.22 (.09)	.74	.69
UDS06	Go down 4 or 5 steps, without handrails	.92	.92		.15			- .47 (.09)	.98	.87
UDS07	Go up 1 flight of stairs, without handrails	.90	.91		.10			- 1.54 (.08)	.91	.88
UDS08	Go down 1 flight of stairs, using handrails	.90	.88		.34			1.42 (.11)	.74	.70
UDS09	Climb stairs carrying little weight	.87	.86		.26			.64 (.10)	.87	.83
UDS10	Go up 3 flights of stairs, using handrails	.88	.88		.22			- .98 (.09)	.75	.79
UDS11	Go down 3 flights of stairs, using handrails	.89	.88		.26			- .29 (.09)	.80	.75
**Body positions** (**BP**)										
BP01	Get into bed†	-	-					-	-	-
BP02	Turn over in bed**	.62	.60			.32		-	-	-
BP03	Sit down on a couch	.83	.83			.23		1.48 (.11)	1.18	.80
BP04	Sitting, bend over to pick something up	.75	.72			.46		0.83 (.10)	1.25	1.23
BP05	Remain seated for 10 minutes without back rest**	.76	.76			.20		-	-	-
BP06	Sit up in bed, being lied down**	.72	.71			.25		-	-	-
BP07	Sit down and stand up from a chair†	-	-					-	-	-
BP08	Stand up from a low, soft couch	.80	.79			.21		- .87 (.09)	1.01	1.16
BP09	Pick up a chair†	-	-					-	-	-
BP10	Reach overhead while standing*	.74	.73			.26		-	-	-
BP11	Turn around while standing**	.70	.69			.23		-	-	-
BP12	Remain standing for 10 minutes	.82	.83			.03		- .15 (.09)	1.14	1.06
BP13	Standing, bend down to pick something up	.79	.77			.47		.02 (.09)	1.12	1.03
BP14	Get up from the floor from lying on your back	.83	.82			.29		−1.96 (.08)	.95	1.03
BP15	Kneel down*	.79	.79			.19		-	-	-
BP16	Get into and out of a car	.76	.76			.20		.54 (.09)	1.10	1.31
**Carrying**, **lifting and pushing** (**CLP**)										
CLP01	Push or pull a large object	.80	.79				.42	.26 (.09)	1.20	1.20
CLP02	Move or drag a bed	.81	.79				.48	.12 (.09)	1.21	1.33
CLP03	Lift 4 or 5 kg from the floor*	.80	.80				.30	-	-	-
CLP04	Turn over a mattress	.81	.80				.34	.36 (.09)	1.21	1.14
CLP05	Change gas bottle*	.82	.81				.39	-	-	-

RSM category thresholds: -1.34 (no difficulty vs. difficulty), 1.34 (difficulty vs. unable to do).

† Items excluded after Testgraf analysis.

* Items excluded after DIF analysis.

** Items excluded (misfit) after Rasch rating scale analysis.

G -- General factor.

g -- Group factor.

*MNSQ* Mean square error.

## Discussion

In this paper, we present the development and validation of a mobility item pool in a sample of 593 older people attending primary health care practices in Spain. Item content was based on ICF mobility indicators, and the item stems and response options concerned difficulty in performing an activity without external help. We examined IRT assumptions, analysed DIF/DBF by sex and calibrated the item pool with the Rasch RSM. No evidence was found of non-uniform DIF but we did observe uniform DIF and DBF. Although the confirmatory factor analysis results satisfied stringent criteria for unidimensionality, the DBF results called this conclusion into question, mainly because with the exception of the Walking domain, all other domains showed DBF, notably the CLP domain. Following the Rasch RSM analysis, 35 items remained in the pool and formed the Late Life Mobility item bank (LLM-IB), which measured with a reliability of 0.90 or higher across the entire spectrum of mobility, except at the extreme end of better function. Lastly, the 35 items were co-calibrated with the PF-10 items.

A noteworthy aspect of this study is that to the best of our knowledge, this is the first time in the literature on patient reported outcomes that DBF has been analysed. To achieve this, in addition to examining DIF according to standard procedure, we also examined augmented DIF at domain level (DBF) using two external criteria as conditioning variables: the PF-10 scale and SPPB. Results of the DIF/DBF analysis with the two external criteria were very similar, suggesting no bias in self-report versus performance-based scales as a method to measure late life mobility: most of the CLP domain items and three BP domain items were flagged for significant DIF. Standard DIF results were less similar to those above, since fewer CLP domain items were identified as presenting significant DIF and there were more items with DIF, significant or nonsignificant, with opposite signs: most of the Walking domain items were negative and all of the CLP domain items were positive. This has also been observed recently during the development of the European Organisation for Research and Treatment of Cancer Physical Function item bank, and the most plausible explanation is that both bundles/domains measure different secondary dimensions [24,25,35].

Although conditioning with an internal criterion such as total score produces DIF values with a trade off between positive and negative values as DIF values are statistically dependent [26], it is interesting that the items which systematically presented opposite values were Walking and CLP items. However, when an external criterion is used as a conditioning variable, statistical dependence disappears [26]. Thus, DIF/DBF analysis using SPPB and PF-10 as conditional variables revealed that CLP measured a secondary dimension that produced significant DIF and DBF, but Walking domain items produced neither DBF nor DIF, with the exception of one item according to SPPB but none according to PF-10. Therefore, standard DIF analysis indicated that Walking items and CLP items measured different domains and DIF/DBF analysis revealed that Walking was the core dimension of the mobility construct and CLP was a secondary dimension that produced DBF. This interpretation, that CLP items measure a secondary dimension of the mobility construct, is also consistent with results from non-psychometric studies, which have reported that gender differences are greater in items in this domain than in other mobility domains [16-21] and that these differences do not disappear after adjustment for important covariables [18,19,51]. These results are also consistent with those found in the fields of geriatric frailty and sarcopenia, where these items are commonly referred to as indicators of strength: walking and strength constitute two separate sub-dimensions of the frailty construct [52,53], and strength is a predictor of mobility decline and is a more intense predictor in men than women [54]. If a secondary dimension produces DIF, the DIF is benign if the dimension is considered part of the construct, but adverse if the secondary dimension is considered a nuisance [25,40]. Therefore, deciding whether the strength domain produces benign DIF or adverse DIF is a theoretical issue, but the data show that the inclusion of strength items increases gender differences in mobility. When validating the LLM-IB, we decided that the strength domain produces benign DBF and we excluded only those items that were consistently flagged for significant DIF.

We used the Rasch RSM to calibrate the item bank and eliminated six of the 41 items that still remained in the item pool, having previously eliminated three for being too easy and four due to DIF. Thus, 35 items remained and constituted the LLM-IB. Most of the Walking and UDS items were retained since they did not present any of the problems observed in the items in the other two domains. We believe that these results help to explain the predominance of walking and going up & down stairs items in the fixed and adaptive physical function measures. Indeed, in the PF-10 and Health Assessment Questionnaire II [55], most of the items are from the Walking or UDS domains. In the new measures, short forms and computer adaptive test applications developed from item banks such as the Patient Reported Outcomes Measurement Information System Physical Function item bank [56] or the Activity Measure for Post Acute Care mobility item bank [6] also produce a predominance of items from the Walking and UDS domains. This occurs even if a content balancing algorithm is introduced to select the first items from the computer adaptive test applications, since the greater wealth of information contained in the Walking and UDS items, calibrated with IRT models which included a discrimination parameter, means that in the end, these achieve greater representation.

The item pool originally contained four response options, but a graphical, non-parametric IRT analysis showed that the number of response options per item should be reduced. We examined two rating scale alternatives, one combining the two intermediate options (“some difficulty” and “much difficulty”) whilst the other combined the two options reflecting greatest difficulty (“much difficulty” and “unable to do”). We chose the first because it was psychometrically better, and because it is common practice to distinguish between difficulty and incapacity in research on the disablement process. Our sample consisted of older people, generally with a poor educational level (reflecting the current cohort of the elderly population in Spain), which alone may explain why a rating scale with three options works better than a rating scale with more [57].

This study has various limitations. Firstly, in the DBF analysis, one of the bundles, CLP domain, contained only five items. Consequently, the idiosyncrasy of these may constitute an alternative explanation to our interpretation based on the validity of five items as a domain measure. However, the items included are among the most common in the literature. In addition, care was taken not to include items that were too demanding and which would thus have favoured men even more. Secondly, although the use of two conditioning variables which are widely accepted as standard physical function and mobility measures is one of the strengths of this analysis, the study lacked a similar standard for the CLP domain: an objective measure of strength would have enhanced the construct validity of the findings. Thirdly, because DIF by age has repeatedly been found for many items in measures of PF, the extrapolation of our results beyond samples of older people is questionable. Finally, our findings are exclusively cross-sectional. We anticipate validating the item bank and several fixed forms with the longitudinal data collected after monitoring the same cohort for 18 months with outcome variables such as mortality, dependency and hospitalization.

## Conclusions

We have designed an item bank in Spanish to measure mobility in older primary care patients which is free from item bias across gender and was calibrated using Rasch RSM. Item bank accuracy and coverage was satisfactory throughout the entire continuum of mobility, with the exception of the upper level of capacity, suggesting the desirability of replenishing the item bank with items that measure at high mobility function level. Furthermore, our results indicate that the walking and going up and down stairs items form the core of the mobility construct whilst strength items form a secondary dimension that produces augmented DIF. These results highlight the desirability of stratifying by domain and weighting domain representation when selecting items to create fixed or adaptive forms of mobility for older people, leaving only strength items marginal. Further research is needed to determine how best to include strength items in a mobility measure, or whether it would be more appropriate to design separate measures for each construct.

## Abbreviations

PF-10: Physical Functioning subscale of the Medical Outcomes Study Short Form-36; IRT: Item Response Theory; ICF: International Classification of Functioning, Disability and Health; DIF: Differential item functioning; DBF: Differential bundle functioning; LLM-IB: Late Life Mobility Item Bank; BP: Changing and maintaining body position mobility domain; CLP: Carrying, lifting and pushing mobility domain; UDS: Going up and down stairs mobility domain; SPPB: Short Physical Performance Battery; ADL: Activity of daily living; RSM: Rating scale model; CFI: Comparative fit index; TLI: Tucker Lewis index; RSMEA: Root-mean-square error of approximation; SRMR: Standardised root mean square residual; SIBTEST: Simultaneous item bias test.

## Competing interests

The authors declare that they have no competing interests.

## Authors’ contributions

JCG conceived the study, performed statistical analysis and drafted the manuscript. JDRP, CLMM, MJCM, LG participated in data collection and management. All authors participated in the design of the study and interpretation of data. All authors read and approved the final manuscript.
